# DIFFERENCES IN CONFORMITY TO MASCULINE NORMS ACROSS AGE COHORTS

**DOI:** 10.1093/geroni/igad104.2682

**Published:** 2023-12-21

**Authors:** Montgomery Owsiany, Erika Fenstermacher, Jeongwi An, Sabine Lohmar, Amy Fiske

**Affiliations:** West Virginia University, Morgantown, West Virginia, United States; West Virginia University, Morgantown, West Virginia, United States; West Virginia University, Morgantown, West Virginia, United States; West Virginia University, Morgantown, West Virginia, United States; West Virginia University, Morgantown, West Virginia, United States

## Abstract

Conformity to masculine norms is associated with worse mental health among men, but little is known about conformity to masculine norms across the lifespan. Much of the literature on this topic focuses on early adulthood, rarely extending past middle age. Research indicates that attaining hegemonic masculinity becomes more difficult as one ages. One study with Australian young and middle-aged men found that conformity to masculine norms was lower in middle-aged cohorts than in younger cohorts. A study of Canadian men aged 55 years and up found that conformity to masculine norms did not significantly differ between those aged 55-69 and those aged 70+ years. The present study assessed differences in conformity to masculine norms across the adult lifespan. It was hypothesized that scores on the conformity to masculine norms inventory (CMNI) would significantly differ across six age groups and would be lower in older cohorts. The mean CMNI score for males (N = 447) was 60.96 (SD = 19.09) and for females (N = 431) was 47.19 (SD = 15.11). In a one-way ANOVA, scores significantly differed between age cohorts for the male (F(5, 441) = 20.83, p < .001) and female samples (F(5,425) = 14.85, p < .001). Tukey’s HSD test for multiple comparisons revealed that CMNI scores were significantly lower in older cohorts compared to younger cohorts in both males and females. Findings highlight the importance of considering age-related differences in the presentation of masculinity. Future studies should investigate whether differences are related to aging or cohort effects.

